# Endothelial Activation and Stress Index Predicts Poor Coronary Collateral Development in Chronic Total Occlusion

**DOI:** 10.3390/jcdd13030124

**Published:** 2026-03-09

**Authors:** Muhammed Ulvi Yalcin, Kadri Murat Gurses, Canan Aydoğan, Sevil Butun, Abdullah Tunçez, Hüseyin Tezcan, Yasin Ozen, Kenan Demir, Nazif Aygul, Mustafa Kirmizigul, Aslihan Merve Toprak Su, Burak Erdogan, Tolgahan Karaman, Bulent Behlul Altunkeser

**Affiliations:** Medical Faculty Hospital, Department of Cardiology, Selçuk University, 42090 Konya, Turkey

**Keywords:** chronic total occlusion, coronary collateral circulation, EASIX, endothelial dysfunction, systemic inflammation

## Abstract

**Background/Objectives:** Coronary collateral circulation (CCC) reduces ischemic damage in patients with chronic total occlusion (CTO), yet collateral development varies considerably among individuals. Endothelial stress and systemic inflammation are key biological processes involved in collateral vessel formation. The Endothelial Activation and Stress Index (EASIX), calculated from lactate dehydrogenase, creatinine, and platelet count, is a simple marker reflecting endothelial dysfunction and inflammatory status. However, evidence regarding its relationship with angiographic coronary collateral development in CTO remains limited. Therefore, this study aimed to evaluate the association between EASIX and CCC grades in patients with CTO. **Methods:** This retrospective study included 186 patients with CTO who underwent coronary angiography. CCC was evaluated using the Rentrop–Cohen classification and categorized as poorly developed (grades 0–1) or well-developed (grades 2–3). Clinical and laboratory data, including EASIX, were collected. Univariate and multivariate binary logistic regression analyses were performed to identify factors associated with poorly developed CCC. EASIX was standardized (z-score), and odds ratios were reported per 1-standard deviation increase. The predictive performance of EASIX was assessed using receiver operating characteristic (ROC) curve analysis. **Results:** Poorly developed CCC was observed in 70 patients (37.6%). Patients with well-developed CCC had significantly lower EASIX values (median 0.44 vs. 0.67, *p* < 0.001) and higher HDL cholesterol levels (*p* = 0.043). Neutrophil-to-lymphocyte ratio was also higher in the poorly developed CCC group (median 2.59 [2.19–3.59] vs. 2.41 [1.59–3.49], *p* = 0.028). In multivariate analysis, standardized EASIX remained independently associated with poorly developed CCC (OR 2.536 per 1-SD increase, 95% CI 1.734–3.710, *p* < 0.001). ROC analysis showed that EASIX provided moderate discrimination for poorly developed CCC (AUC 0.718), with 72.9% sensitivity and 62.1% specificity at a cutoff of >0.51. **Conclusions:** Higher EASIX values were independently associated with poorly developed CCC in patients with CTO. These findings support a link between systemic endothelial stress and impaired collateral vessel formation. EASIX may serve as a simple, practical, and low-cost biomarker to support risk stratification in CTO patients; however, prospective studies are needed to confirm these results and clarify clinical implications.

## 1. Introduction

Stable coronary artery disease remains a major cause of morbidity and mortality worldwide, and contemporary management includes risk-factor control, optimal medical therapy, and physiology-guided revascularization strategies according to the clinical likelihood and ischemic burden [1]. Current evidence-based approaches emphasize individualized treatment decisions integrating clinical presentation, diagnostic testing, and anatomical assessment [1]. Complex coronary lesions within stable coronary artery disease may present specific diagnostic and therapeutic challenges requiring careful evaluation and tailored management strategies [1].

Chronic total occlusion (CTO) represents one of the most advanced forms of coronary artery disease (CAD) and is defined by complete coronary obstruction with Thrombolysis in Myocardial Infarction (TIMI) grade 0 flow persisting for more than three months [2]. CTO lesions are frequently encountered during coronary angiography and are associated with a substantial ischemic burden, impaired ventricular function, and adverse clinical outcomes if left untreated [3]. In this setting, myocardial perfusion distal to the occluded segment depends largely on the presence and functional capacity of coronary collateral circulation (CCC) [2,3].

Well-developed CCC confers important protective effects, including attenuation of ischemic injury, preservation of left ventricular systolic function, and improved clinical outcomes [4,5]. However, despite similar ischemic exposure, marked interindividual variability exists in collateral vessel development among patients with CTO [6,7], suggesting that collateral formation is influenced by factors beyond ischemia alone.

Coronary collateral development is a complex process involving arteriogenesis, angiogenesis, endothelial activation, inflammatory signaling, and shear stress–mediated vascular remodeling [8]. Endothelial dysfunction may impair these adaptive mechanisms, limiting effective collateral formation [7,9]. Accordingly, systemic inflammation, oxidative stress, metabolic dysregulation, and hematologic abnormalities have been implicated as important modulators of CCC development [10].

Several readily available laboratory indices reflecting inflammation and endothelial dysfunction have been associated with impaired collateral development [11,12,13,14,15]. The Endothelial Activation and Stress Index (EASIX), calculated from lactate dehydrogenase, serum creatinine, and platelet count, integrates markers of tissue injury, microvascular dysfunction, and endothelial activation, and has emerged as a surrogate measure of systemic endothelial stress [16,17]. EASIX has demonstrated prognostic relevance in various cardiovascular conditions [17,18,19,20], yet its relationship with coronary collateral development remains poorly defined.

Accordingly, this study aimed to evaluate the association between EASIX and angiographic coronary collateral development in patients with CTO and to explore clinical and laboratory determinants of collateral vessel formation in this high-risk population.

## 2. Materials and Methods

### 2.1. Study Design and Patient Population

This retrospective observational study included consecutive patients who underwent diagnostic coronary angiography for stable CAD between January 2020 and November 2025 at a tertiary care center. Patients were eligible for inclusion if they were found to have at least one CTO lesion in a major epicardial coronary artery (CA). CTO was defined as a complete luminal obstruction with TIMI grade 0 flow and an estimated occlusion duration of greater than three months, based on clinical history, prior angiographic data, or electrocardiographic and imaging findings.

Patients were excluded if they had experienced an acute coronary syndrome, percutaneous coronary intervention, or CA bypass graft surgery within the preceding three months, in order to minimize the confounding effects of acute ischemia and procedural interventions on laboratory parameters and collateral assessment. Additional exclusion criteria included moderate renal dysfunction (estimated glomerular filtration rate < 60 mL/min/1.73 m^2^), active or chronic inflammatory or infectious diseases, known malignancy, significant valvular heart disease, left ventricular ejection fraction below 40%, anemia (hemoglobin < 13 g/dL in men and <12 g/dL in women), hematologic disorders, or a history of blood transfusion within the previous three months.

Ethics approval was obtained retrospectively from the Ethics Committee of Selçuk University Faculty of Medicine (approval number: 2025/788), and the investigation was conducted in accordance with the principles outlined in the Declaration of Helsinki. Due to the retrospective nature of the study, the requirement for informed consent was waived.

### 2.2. Clinical and Laboratory Data Collection

Demographic characteristics, cardiovascular risk factors (including hypertension, diabetes mellitus (DM), hyperlipidemia, and smoking status), comorbid conditions, and medication use were obtained from electronic medical records. Blood pressure measurements and body mass index were recorded at the time of hospital admission.

Peripheral venous blood samples were collected after an overnight fast and prior to coronary angiography. Complete blood count analyses were performed using EDTA-anticoagulated samples analyzed on an automated hematology analyzer. Parameters recorded included hemoglobin concentration, total leukocyte count, and platelet count.

Biochemical analyses were performed using standard automated laboratory techniques and included measurements of serum creatinine, LDH, total cholesterol, triglycerides, low-density lipoprotein (LDL) cholesterol, and high-density lipoprotein (HDL) cholesterol. Laboratory analyses were performed using standardized methods throughout the study period.

EASIX was calculated for each patient using the following formula [17]:

**EASIX = [LDH (U/L) × creatinine (mg/dL)]/platelet count (10^9^/L)**

### 2.3. Angiographic Assessment

Angiographic acquisitions were performed using standard catheter sizes and routine contrast injection techniques according to institutional practice. Collateral grading was performed independently by two experienced interventional cardiologists. Interobserver agreement for Rentrop grading was high (Cohen’s κ = 0.86), indicating excellent reproducibility. Discrepant cases were resolved by consensus review.

CCC was graded according to the Rentrop–Cohen classification system [21]:

Grade 0, no visible collateral flow;
Grade 1, filling of side branches without visualization of the epicardial segment;
Grade 2, partial filling of the epicardial segment;
Grade 3, complete filling of the epicardial segment.

For statistical analysis, patients were categorized into two groups: poorly developed CCC (Rentrop grades 0–1) and well-developed CCC (Rentrop grades 2–3).

### 2.4. Statistical Analysis

Statistical analyses were performed using SPSS software version 21.0 (SPSS Inc., Chicago, IL, USA). The normality of continuous variables was assessed using the Kolmogorov–Smirnov test. Continuous variables were expressed as mean ± standard deviation or median with interquartile range, as appropriate, while categorical variables were presented as frequencies and percentages.

Comparisons between groups were conducted using the independent samples *t*-test or Mann–Whitney U test for continuous variables and the chi-square test for categorical variables. Univariate binary logistic regression analysis was performed to identify variables associated with poorly developed CCC. To improve interpretability, EASIX was standardized (z-score), and odds ratios were reported per 1-standard deviation increase in EASIX. Variables with a *p*-value ≤ 0.20 in univariate analysis were entered into a multivariate logistic regression model to identify factors independently associated with poorly developed CCC. To minimize model overfitting given the limited number of outcome events, the multivariate model was constructed using a restricted number of clinically relevant covariates. Although LDH, serum creatinine, and platelet count met this criterion, they were excluded from the multivariate model as individual variables because they are the constitutive components of the EASIX score. Including these parameters alongside the EASIX score would lead to multicollinearity and potential overfitting of the regression model. This strategy improves model interpretability and allows the composite index to reflect overall endothelial stress burden rather than isolated component effects. Therefore, the multivariate model was constructed using the EASIX score and other non-component variables (e.g., HDL cholesterol and neutrophil-to-lymphocyte ratio) that met the *p*-value threshold. Seventy patients had poorly developed coronary collateral circulation and were considered outcome events. The final multivariate model included three independent variables, corresponding to an events-per-variable (EPV) ratio of approximately 23. This EPV exceeds the commonly recommended minimum threshold of 10 events per variable and supports the stability and reliability of the regression model [22]. Receiver operating characteristic (ROC) curve analysis was used to evaluate the discriminative ability of EASIX for identifying poorly developed CCC. The optimal cutoff value was determined using the Youden index. A two-sided *p* value < 0.05 was considered statistically significant.

## 3. Results

A total of 186 patients with CTO were included in the analysis. According to the degree of CCC, 70 patients (37.6%) were classified as having poorly developed CCC (Rentrop grades 0–1), while 116 patients (62.4%) had well-developed CCC (Rentrop grades 2–3). Baseline clinical and demographic characteristics of the study population are summarized in Table 1.

Baseline clinical characteristics were similar between the groups, including age, BMI, sex distribution, smoking status, diabetes mellitus (DM), and hypertension (all *p* > 0.05). Hemodynamic parameters and LVEF were also comparable.

The anatomical distribution of the occluded CA (left anterior descending, circumflex, or right CA) did not differ significantly between the groups. In addition, the use of beta-blockers, renin–angiotensin system blockers, and statins was similar in both groups (Table 1).

### 3.1. Laboratory Findings

Laboratory parameters according to collateral status are presented in Table 2. Patients with well-developed CCC had significantly higher HDL cholesterol levels compared with those with poorly developed CCC (median 40 [35–45] vs. 38 [35–41] mg/dL, *p* = 0.043).

LDH levels were significantly lower in the well-developed CCC group (median 129 [107–149] vs. 173 [137–184] U/L, *p* < 0.001). Serum creatinine levels were also lower in well-developed CCC (median 0.91 [0.78–1.00] vs. 0.97 [0.80–1.20] mg/dL, *p* = 0.017).

**Figure 1:**
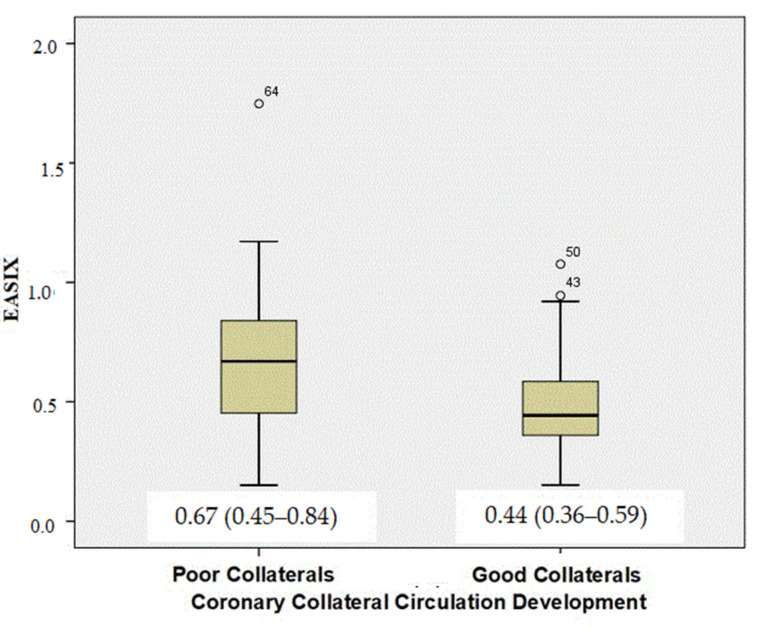
Comparison of the Endothelial Activation and Stress Index (EASIX) according to the degree of coronary collateral circulation. Values are presented as median (interquartile range).

The EASIX score was significantly higher in patients with poorly developed CCC (median 0.67 [0.45–0.84] vs. 0.44 [0.36–0.59], *p* < 0.001) (Figure 1). Neutrophil-to-lymphocyte ratio was significantly higher in the poorly developed CCC group (median 2.69 [2.29–3.69] vs. 2.31 [1.49–3.50], *p* = 0.002). No significant differences were observed between the groups regarding LDL cholesterol, triglyceride levels, hemoglobin concentration, white blood cell count, or monocyte-to-HDL cholesterol ratio. Platelet count showed a trend toward significance (*p* = 0.053).

### 3.2. Logistic Regression and ROC Analysis

In univariate logistic regression analysis, higher HDL cholesterol levels were associated with a lower odds of poorly developed CCC (OR 0.948, 95% CI 0.900–0.997, *p* = 0.039). Neutrophil-to-lymphocyte ratio showed a non-significant trend toward association with poorly developed CCC (OR 1.110, 95% CI 0.958–1.286, *p* = 0.163). Standardized EASIX (per 1-SD increase) was strongly associated with poorly developed CCC (OR 2.537, 95% CI 1.751–3.675, *p* < 0.001). Elevated LDH and serum creatinine levels were also significantly associated with poor collateral development in univariate analysis (Table 3). Lactate dehydrogenase and serum creatinine were excluded from the multivariate model due to their incorporation into the EASIX score, in order to avoid multicollinearity and potential overfitting of the regression model.

In the multivariate logistic regression model, standardized EASIX remained independently associated with poor coronary collateral development (OR 2.551 per 1-SD increase, 95% CI 1.743–3.734, *p* < 0.001), whereas the association between HDL cholesterol and collateral development did not retain statistical significance (*p* = 0.060). Neutrophil-to-lymphocyte ratio was not independently associated with collateral status (*p* = 0.127).

**Figure 2:**
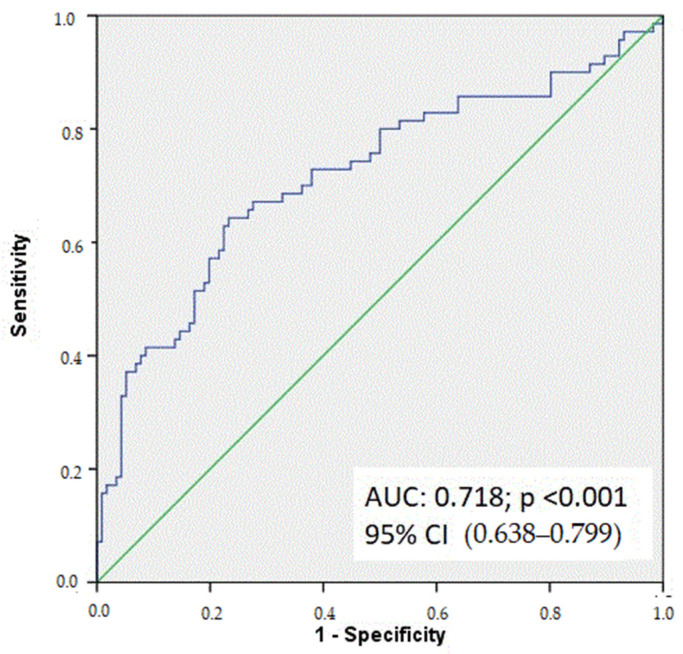
Receiver operating characteristic (ROC) curve of the Endothelial Activation and Stress Index (EASIX) for predicting poorly developed coronary collateral circulation. An EASIX value > 0.51 was associated with poorly developed CCC, with a sensitivity of 72.9% and specificity of 62.1% (AUC = 0.718; 95% CI: 0.638–0.799). The optimal cutoff value was determined using the Youden index.

ROC curve analysis demonstrated that EASIX showed moderate discriminative ability for identifying poorly developed CCC with an area under the curve (AUC) of 0.718 (95% CI: 0.638–0.799). An EASIX cutoff value >0.51 was associated with poorly developed CCC, with a sensitivity of 72.9% and a specificity of 62.1% (Figure 2).

## 4. Discussion

In this retrospective study of patients with CTO, higher EASIX values were independently associated with poorly developed CCC. This association persisted after adjustment for relevant clinical and laboratory variables, suggesting that a greater systemic endothelial stress burden is associated with impaired adaptive collateralization rather than ischemic exposure alone. These findings extend existing evidence by highlighting the potential role of composite endothelial stress indices in characterizing vascular vulnerability in CTO. Coronary collateral development is a biologically complex process that depends on coordinated arteriogenesis and endothelial-mediated vascular remodeling in response to ischemia and shear stress. Endothelial dysfunction impairs nitric oxide bioavailability, disrupts mechanotransduction, and promotes oxidative stress, all of which are essential for collateral vessel formation and maturation [10,23]. Our findings support the concept that a systemic milieu characterized by increased endothelial stress and inflammation may be associated with impaired collateral vessel formation, even in the presence of similar ischemic stimuli.

In particular, LDH, a non-specific marker of tissue injury, may increase in response to ongoing myocardial ischemia and cellular injury. Therefore, elevated EASIX values may partly reflect the consequences of insufficient collateral circulation rather than a primary determinant of collateral development. Accordingly, the present cross-sectional retrospective design does not allow causal inference, and EASIX should be interpreted as a biomarker associated with collateral status rather than direct evidence of endothelial stress impairing collateral formation. Reverse causation cannot be excluded. Prospective longitudinal studies are needed to clarify the temporal relationship between EASIX and coronary collateral development.

The EASIX score, calculated using lactate dehydrogenase, serum creatinine, and platelet count, was originally introduced as a prognostic marker in patients undergoing hematopoietic stem cell transplantation, reflecting endothelial injury, capillary leak, and systemic inflammatory stress [24]. More recently, EASIX has been proposed as a surrogate marker of endothelial dysfunction and microvascular damage in non-hematologic conditions, including cardiovascular disease [17,19,25,26,27,28]. Elevated EASIX values have been associated with adverse outcomes in patients with CAD, heart failure, and acute coronary syndromes, independent of traditional cardiovascular risk factors [18,19,29,30].

In the context of CAD, preserved endothelial integrity is essential for maintaining vascular tone, regulating inflammatory cell adhesion, and facilitating adaptive vascular remodeling [31]. Elevated LDH levels may reflect increased cellular turnover and tissue injury in the setting of chronic ischemia and systemic inflammation [32]. Serum creatinine serves as a marker of renal and microvascular dysfunction, both closely linked to endothelial impairment [33]. Platelet count reflects the balance between thrombogenic activity and endothelial regulation [34]. The integration of these parameters into a single composite index such as EASIX may therefore better capture the overall endothelial stress burden than individual laboratory markers alone.

The observed association between higher EASIX scores and poorly developed CCC is biologically plausible. Endothelial dysfunction has been shown to reduce nitric oxide bioavailability, increase oxidative stress, and impair shear stress–mediated mechanotransduction, all of which are critical for collateral vessel recruitment and maturation [31]. Nitric oxide plays a central role in arteriogenesis by promoting vasodilation, inhibiting smooth muscle cell proliferation, and facilitating endothelial cell migration [35]. Thus, higher EASIX values may reflect an unfavorable endothelial environment that is less conducive to effective collateral vessel development, rather than directly inhibiting arteriogenesis.

Inflammation represents another important determinant of collateral development. While a controlled inflammatory response is necessary for arteriogenesis, excessive or persistent inflammation may exert unfavorable effects. Pro-inflammatory cytokines such as tumor necrosis factor-alpha, interleukin-6, and C-reactive protein have been reported to correlate inversely with coronary collateral formation, suggesting that an adverse inflammatory milieu may hinder effective vascular remodeling [36,37]. Given that EASIX indirectly reflects systemic inflammatory stress, its association with poor collateral development may partly be mediated through an adverse inflammatory milieu.

In the present study, ROC curve analysis demonstrated that EASIX had a moderate ability to discriminate patients with poorly developed CCC, with an area under the curve of 0.718. The moderate discriminative performance of EASIX should be interpreted in the context of its simplicity and routine availability. While EASIX is not intended as a standalone diagnostic tool, its ability to identify patients at risk of poor collateralization may be clinically useful as part of a broader risk stratification.

Consistent with previous reports, higher EASIX values have also been associated with adverse outcomes in acute ischemic settings, including increased short-term mortality in patients with acute myocardial infarction [30]. These observations suggest that EASIX may reflect a broader vascular vulnerability phenotype relevant to both chronic ischemic adaptation and acute ischemic injury.

Although HDL cholesterol levels were modestly higher in patients with well-developed CCC, HDL did not remain independently associated with collateral development in multivariate analysis. HDL is known to exert protective effects on endothelial function through antioxidant and anti-inflammatory mechanisms [38,39]; however, HDL functionality—which was not assessed in the present study—may be more relevant to vascular remodeling than absolute HDL cholesterol levels. This may explain the attenuation of its association after adjustment for systemic endothelial stress as reflected by EASIX.

Although platelet count showed a borderline association with collateral status (*p* = 0.053), neutrophil-to-lymphocyte ratio was significantly higher in the poorly developed CCC group (*p* = 0.002); however, it did not remain independently associated with collateral status in multivariate analysis. This finding suggests that EASIX may capture a broader endothelial stress phenotype beyond isolated inflammatory markers.

From a clinical perspective, identifying patients with poor coronary collateralization is relevant in CTO, as inadequate collateral flow may influence symptom burden, ischemic tolerance, and revascularization strategies. Although the present findings are hypothesis-generating, EASIX, derived from routine laboratory parameters, may serve as a practical, low-cost, and cost-effective biomarker to support risk stratification in CTO patients and to may help identify individuals who could be at higher risk of inadequate collateralization who may benefit from closer clinical surveillance and optimization of medical therapy.

Several limitations of this study should be acknowledged. First, the retrospective, single-center design limits causal inference and reduces the generalizability of our findings. Second, coronary collateral circulation (CCC) was evaluated using the semi-quantitative angiographic Rentrop grading system, which is based on contrast filling and may not accurately reflect functional collateral flow capacity. Functional measurements such as collateral flow index or pressure-derived collateral assessment would provide a more precise evaluation of collateral function. Therefore, the clinical applicability of angiographic collateral grading may be limited compared with physiological collateral assessment. Third, important anatomical and ischemic determinants of collateral development, including occlusion length, distal vessel quality, proximal cap characteristics, myocardial viability, and CTO complexity scores such as the J-CTO score, were not systematically available in this retrospective dataset and therefore could not be included in the analysis. These factors may influence collateral formation and represent potential sources of residual confounding; thus, they should be addressed in future prospective studies. Fourth, patients with moderate renal dysfunction were excluded to reduce confounding; therefore, our results may not be generalizable to patients with chronic kidney disease. Fifth, the components of EASIX may be affected by non-cardiovascular conditions, which could have introduced confounding despite the applied exclusion criteria. In addition, the relatively strict exclusion criteria (including reduced renal function, anemia, reduced left ventricular ejection fraction, and inflammatory conditions) may have resulted in a selected CTO population that does not fully reflect real-world clinical practice, thereby limiting external validity. Although patients with moderate renal dysfunction were excluded, small but statistically significant differences in creatinine and LDH levels remained between groups. These laboratory differences may reflect variations in ischemic burden, subclinical renal impairment, metabolic conditions, or other unmeasured biological factors. Therefore, EASIX in this context may partially represent underlying disease severity or physiological stress rather than a specific endothelial mechanism. Finally, residual confounding cannot be excluded, and the cross-sectional nature of the analysis precludes assessment of temporal changes in EASIX and collateral development over time.

In conclusion, elevated EASIX scores were independently associated with poorly developed CCC in patients with CTO. These findings suggest a potential association between systemic endothelial stress and impaired adaptive collateralization. EASIX may represent a simple and accessible biomarker for vascular risk stratification in CTO, although prospective and mechanistic studies are required to confirm causality and clinical utility.

## Figures and Tables

**Table 1:** Comparison of clinical and demographic characteristics according to the degree of coronary collateral circulation development.

Variable	Poorly Developed CCC (*n* = 70)	Well-Developed CCC (*n* = 116)	*p* Value
Age, years	60 (54–70)	62 (55–71)	0.432
Body mass index, kg/m^2^	27.74 (25.45–30.47)	27.66 (26.15–30.43)	0.818
Male sex, *n* (%)	55 (78.6)	99 (85.3)	0.238
Smoking, *n* (%)	34 (48.6)	62 (53.4)	0.547
Diabetes mellitus, *n* (%)	25 (35.7)	47 (40.5)	0.538
Hypertension, *n* (%)	41 (58.6)	76 (65.5)	0.352
Systolic blood pressure, mmHg	120 (110–130)	110 (110–120)	0.166
Diastolic blood pressure, mmHg	70 (70–80)	70 (70–80)	0.919
Heart rate, beats/min	75 (70–80)	75 (68–80)	0.173
Left ventricular ejection fraction, %	55 (50–60)	55 (48–60)	0.522
**Location of occluded artery**			0.415
– Left anterior descending artery, *n* (%)	22 (31.4)	45 (38.8)	
– Circumflex artery, *n* (%)	14 (20.0)	26 (22.4)	
– Right coronary artery, *n* (%)	34 (48.6)	45 (38.8)	
**Medication use**			
Beta-blocker, *n* (%)	45 (64.3)	77 (66.4)	0.874
Renin–angiotensin system blockers, *n* (%)	34 (48.6)	63 (54.3)	0.454
Statin, *n* (%)	65 (92.9)	102 (87.9)	0.328

**Abbreviations:** CCC, Coronary Collateral Circulation.

**Table 2:** Comparison of laboratory characteristics according to the degree of coronary collateral circulation development.

Variable	Poorly Developed CCC (*n* = 70)	Well-Developed CCC (*n* = 116)	*p* Value
LDL cholesterol, mg/dL	89 (63–118)	83 (66–121)	0.507
HDL cholesterol, mg/dL	38 (35–41)	40 (35–45)	0.043
Triglycerides, mg/dL	151 (109–210)	134 (97–207)	0.353
Hemoglobin, g/dL	13.74 ± 2.02	13.55 ± 1.89	0.523
White blood cell count, ×10^3^/µL	7.81 (6.59–9.57)	8.27 (7.01–10.07)	0.258
Platelet count, ×10^9^/L	235.09 ± 48.23	249.78 ± 50.80	0.053
Neutrophil-to-lymphocyte ratio	2.59 (2.19–3.59)	2.41 (1.59–3.49)	0.028
Monocyte-to-HDL cholesterol ratio	0.0158 (0.0114–0.0213)	0.0154 (0.0117–0.0194)	0.664
Lactate dehydrogenase, U/L	173 (137–184)	129 (107–149)	<0.001
Creatinine, mg/dL	0.97 (0.80–1.20)	0.91 (0.78–1.00)	0.017
EASIX score	0.67 (0.45–0.84)	0.44 (0.36–0.59)	<0.001

**Abbreviations:** CCC, Coronary Collateral Circulation; EASIX, Endothelial Activation and Stress Index; HDL, high-density lipoprotein; LDL, low-density lipoprotein.

**Table 3:** Binary logistic regression analysis for factors associated with poorly developed CCC.

Variable	Univariate OR (95% CI)	*p* Value	Multivariate OR (95% CI)	*p* Value
Age, years	0.985 (0.957–1.015)	0.329	–	–
Body mass index	0.994 (0.929–1.064)	0.859	–	–
Diabetes mellitus	0.816 (0.442–1.506)	0.515	–	–
Hypertension	0.744 (0.404–1.370)	0.343	–	–
LDL cholesterol	1.002 (0.994–1.010)	0.656	–	–
HDL cholesterol	0.948 (0.900–0.997)	0.039	0.948 (0.896–1.003)	0.064
Triglycerides	1.001 (0.997–1.005)	0.543	–	–
White blood cell count	0.924 (0.808–1.056)	0.245	–	–
Neutrophil-to-lymphocyte ratio	1.152 (0.960–1.383)	0.127	1.146 (0.960–1.368)	0.131
Platelet count	0.994 (0.988–1.000)	0.055	–	–
Lactate dehydrogenase	1.028 (1.017–1.039)	<0.001	–	–
Creatinine	16.020 (3.096–82.909)	0.001	–	–
EASIX (per 1-SD increase)	2.537 (1.751–3.675)	<0.001	2.536 (1.734–3.710)	<0.001

EASIX was standardized (z-score) and odds ratios are reported per 1-SD increase. **Abbreviations:** CCC, Coronary Collateral Circulation; EASIX, Endothelial Activation and Stress Index; HDL, high-density lipoprotein; LDL, low-density lipoprotein.

## Data Availability

The data presented in this study are available from the corresponding author upon reasonable request.
